# Reduction of the molecular hamiltonian matrix using quantum community detection

**DOI:** 10.1038/s41598-021-83561-x

**Published:** 2021-02-18

**Authors:** Susan M. Mniszewski, Pavel A. Dub, Sergei Tretiak, Petr M. Anisimov, Yu Zhang, Christian F. A. Negre

**Affiliations:** 1 Computer, Computational and Statistical Sciences Division, Los Alamos National Laboratory, Los Alamos, NM 87545 USA; 2 Chemistry Division, Los Alamos National Laboratory, Los Alamos, NM 87545 USA; 3 Theoretical Division, Los Alamos National Laboratory, Los Alamos, NM 87545 USA; 4 Accelerator Operations and Technology Division, Los Alamos National Laboratory, Los Alamos, NM 87545 USA

**Keywords:** Physical chemistry, Mathematics and computing, Quantum information

## Abstract

Quantum chemistry is interested in calculating ground and excited states of molecular systems by solving the electronic Schrödinger equation. The exact numerical solution of this equation, frequently represented as an eigenvalue problem, remains unfeasible for most molecules and requires approximate methods. In this paper we introduce the use of *Quantum Community Detection* performed using the D-Wave quantum annealer to reduce the molecular Hamiltonian matrix in Slater determinant basis without chemical knowledge. Given a molecule represented by a matrix of Slater determinants, the connectivity between Slater determinants (as off-diagonal elements) is viewed as a graph adjacency matrix for determining multiple communities based on modularity maximization. A gauge metric based on perturbation theory is used to determine the lowest energy cluster. This cluster or sub-matrix of Slater determinants is used to calculate approximate ground state and excited state energies within chemical accuracy. The details of this method are described along with demonstrating its performance across multiple molecules of interest and bond dissociation cases. These examples provide proof-of-principle results for approximate solution of the electronic structure problem using quantum computing. This approach is general and shows potential to reduce the computational complexity of post-Hartree–Fock methods as future advances in quantum hardware become available.

## Introduction

At this stage in quantum computing, it is useful to demonstrate the application of quantum algorithms to real-world problems even at small scale where classical solutions are available, though may be approximate. This allows for verification and validation, and helps us prepare for the larger-scale problems with unknown results on future quantum hardware. Noisy Intermediate-Scale Quantum (NISQ) technology has provided us the opportunity to explore new approaches to simulate chemistry and physics^1^. By taking advantage of quantum–mechanical effects, NISQ devices promise to improve solutions, provide new pathways to solutions and even solve the problems that are intractable on current classical computers. In addition, quantum formulations of complex network algorithms have gained interest in quantum information science^2^. The cross-disciplinary approach of combining network science and quantum computing has provided new ways to solve chemistry and physics problems that can be framed as graphs. In this work we explore reducing the Hamiltonian matrix for electronic structure calculations using *Quantum Community Detection*^3,4^ on the D-Wave Quantum Annealer. The approach does not rely on any prior knowledge about the chemical system of interest and is based on a matrix formulation only.

Computational chemistry aims to solve the Schrödinger equation numerically for many-body quantum systems of interest such as molecules or solid-state materials. For the vast majority of applications, we are interested in solving the time-independent, non-relativistic form of this equation for electrons by treating nuclei as classical point charges (using the Born–Oppenheimer approximation). The resulting electronic Hamiltonian can be solved exactly within the space spanned by the finite one-electron orbital basis set or a numerical grid. The corresponding method is known as full configuration interaction (FCI) or the diagonalization of the Hamiltonian matrix in the basis of Slater determinants (SDs). For an *N*-electron molecule, the HF states ${\Psi _0}$ form the lowest-energy SD. Other SDs can be described by showing their difference from the HF determinant $|\Psi _0\rangle$, including singly excited SDs $|\Psi ^a_i\rangle$ (which differ from $|\Psi _0\rangle$ by exchanging an occupied with an unoccupied orbital), doubly excited SDs $|\Psi ^{ab}_{ij}\rangle$, and up to *N*-tuple excited determinants $|\Psi ^{abc\cdots }_{ijk\cdots }\rangle$. These SDs form a complete basis of the problem in Hilbert space accounting for the exchange and correlation energy of the multielectronic system. The diagonalization of the resulting FCI matrix with elements $\langle \Psi _m|H|\Psi _n\rangle$, where *m*/*n* goes over all possible SDs, provides an unambiguous standard with which to compare more approximate methods. Since FCI scales factorially with the number of electrons and spin-orbitals, its practical implementation is limited to very small molecules/basis sets. For example, molecular dicarbon (C_2_) in aug-cc-pVTZ basis (with 12 electrons to be distributed on 184 spin-orbitals) is one of the largest molecules ever simulated by means of FCI using the Oak Ridge National Lab Cray-X1 supercomputer^5^. To overcome these limitations, various approximate solutions have been developed by either truncating the problem in a reduced user-defined active space (e.g. multiconfigurational self-consistent methods such as the complete active space self-consistent field (SCF) method (CASSCF)^6–8^) or in a few SDs by employing Configurational Interaction (CI) or Coupled Cluster (CC) approximations^9^. Examples of the truncation methods include CI + single excitations (CIS), CI + single and double excitations (CISD), CI + single, double, and triple excitations (CISDT), CI + single, double, triple, and quadruple excitations (CISDTQ), and coupled cluster single-double perturbative triple (CCSD(T)), to name a few. In their appreciable level of flavor, they scale as ${\mathcal {O}}(N^{6-10})$ and are also applicable only to few atom systems. The Hartree–Fock (HF) mean-field approximation reduces the problem to a single electron moving in an average field of others with ${\mathcal {O}}(N^4)$ complexity, and serves as an upper bound for an energy representing an uncorrelated system of electrons.

Quantum chemistry is regarded as one of the first disciplines that will benefit from quantum computing. Predicting the properties of atoms and molecules will become one of the most important applications of quantum computers^10–12^. Specifically, quantum computing promises to revolutionize quantum simulations of molecules and materials by bringing down the intractable exponential cost on classical computers [ ${\mathcal {O}}(\alpha ^N)$] to a polynomial scaling on quantum computers. This can be achieved by using either of the two available forms of quantum computation: gate-based quantum computing and adiabatic quantum computing. Current quantum computers are hardware-limited in both the number and quality of qubits, qubit connectivity, presence of high noise levels, and the need for full error-correction^13^. Noisy gate-based quantum computers are currently available at the scale of ~50 to 70 qubits Adiabatic quantum computers are available in the form of quantum annealers, such as the D-Wave 2000Q (with ~2048 qubits) and the new D-Wave Advantage machine (with ~5000 qubits). Quantum annealing architectures require a problem to be framed as an Ising Model or quadratic unconstrained binary optimization (QUBO) and allow for slow continuous transformation of an initial Hamiltonian to a target Hamiltonian settling into a low energy state. Qubit count and limited connectivity between qubits can restrict the size of the problem that can be run directly on the hardware, necessitating the use of quantum–classical algorithms when larger. Gate-based quantum architectures require that problems be transformed into a circuit of reversible gates applied to a given set of qubits prior to execution. The use of shallow depth circuits and variational quantum–classical algorithms mitigate the effects of gate fidelity, short coherence time, and noisy qubits. The D-Wave quantum annealer exhibits an even shorter coherence time, though sufficient for solving optimization problems.

Solutions to electronic structure problems have been demonstrated for small molecules on gate-based quantum computers^14–17^ and the D-Wave 2000Q quantum annealer^18–20^. Polynomial scaling has not yet been demonstrated for the electronic structure problem on a quantum annealer. However, as we show in this work, the most advanced D-Wave 2000Q quantum annealer is well suited to solve combinatorial optimization problems, which can be used to reduce the complexity of the electronic structure problem. In this work we reduce the molecular Hamiltonian on a quantum annealer and then calculate the approximate ground and/or excited state energies on a classical computer through diagonalization. The advantages of solving the graph partitioning and community detection problem on a Quantum Annealer were recently demonstrated in^3,4^. The smaller sub-matrices resulting from the clusters of SDs are then candidates for the best low energy solution. The one containing the lowest energy SD (i.e. the HF SD) or resulting in the lowest gauge metric (described later) is the cluster that provides the best approximation of the original system. The energy is calculated through diagonalization on a classical computer. This typically results in energy many times within chemical accuracy ($\le$ 1 kcal/mol or 1.6e−03 Hartrees).

A quantum annealer, such as the D-Wave 2000Q^21,22^, is able to solve graph problems as combinatorial optimization formulated as an Ising model or QUBO problem. Quantum annealing uses the quantum-mechanical effects of tunneling, superposition and entanglement^23^ to minimize and sample from energy-based models. Evidence that these effects play a useful role in the processing have been discussed in^24–26^. The following objective function in Ising Model form is minimized.

1 $$\begin{aligned} O(\mathbf{h},J,s ) = \sum \limits _{i}h_{i}s_i + \sum \limits _{i<j}J_{ij}s_is_j \end{aligned}$$

where $s_{i} \in$ $\{-1,+1\}$ encodes the binary results; $h_{i}$ and $J_{ij}$ represent the weights on the qubits and strengths on the couplers between qubits of the problem Hamiltonian. The D-Wave quantum computer is composed of qubits with sparse connectivity as a fixed sparse graph, known as a *Chimera* graph. Qubits are in a “superposition” state (both a “− 1” and a “+ 1” simultaneously) during the annealing process. Once the annealing is done, each qubit settles into an Ising spin value $\in$ ${-1,+1}$, resulting in a low-energy ground state. Due to sparse connectivity, a logical variable maps to a chain of qubits^26^ in the D-Wave quantum processing unit (QPU).

Quantum annealers are useful for tackling NP-hard complex problems including optimization, machine learning and sampling. Maximization can also be solved by using the negative of Eq. (1) as the objective function. The QUBO formulation where variables $x_i$ take values of either 0 or 1 is an alternative representation. The QUBO and Ising Model are related by the following linear transformation: $s = 2x - 1$.

*Quantum Community Detection* is Community Detection^27,28^ formulated as a QUBO problem and is run on the D-Wave quantum annealer. The community detection algorithm is an unsupervised machine learning technique that allows for the discovery of network substructure as tightly knit clusters or communities. The quantum version of this algorithm as a QUBO problem running on a quantum annealer uses superposition to explore the design space and settles into a low-energy solution. The molecule Hamiltonian matrix *W* is treated as an adjacency matrix *A* for a graph with weighted edges.

$$\begin{aligned} A_{ij}&= 0, {i = j} \\ A_{ij}&= |{W_{ij}}|, {i \ne j} \end{aligned}$$

The modularity matrix *B* is constructed from the adjacency matrix *A* and degree of each node (number of edges connected to a node), $g_i$, where, $2m = \sum _i g_i$.

$$\begin{aligned} B_{ij} = A_{ij} - \frac{g_ig_j}{2m} = A_{ij} - \frac{g_ig_j}{\sum _{i}g_{i}} \end{aligned}$$

The objective function for the optimal modularity *Q* as a QUBO is maximized as shown in Eq. (2). The elements of *x* are the solution and are binary, where $x_i$ is 0 or 1. Details of the mathematical formulation for multiple communities have been developed previously and are available^4^. When solving for multiple communities, a *one hot encoding* or super-node concept is used with unary encoding. The objective function is shown as the following.

2 $$\begin{aligned} Q(x) = max(x^TBx) \end{aligned}$$

The D-Wave 2000Q is limited in the number of variables that can be embedded and solved directly on the hardware. Therefore, the largest fully connected graph that can be embedded is 65. A hybrid quantum-classical approach is required to address larger numbers of variables as nodes (here representing SDs)^13^. We use the D-Wave developed *qbsolv* classical solver^29^ to orchestrate the QUBO solution process between the central processing unit (CPU) and QPU. Global minimization is performed by *qbsolv*, making multiple calls to the D-Wave to solve sub-QUBOs. This is followed by local minimization using tabu search. The D-Wave Ocean application programming interface (API) or the command line is used to initiate this process. A low-energy solution is returned as a bitstring of zeros and ones that requires translation based on the problem representation.

**Figure 1:**
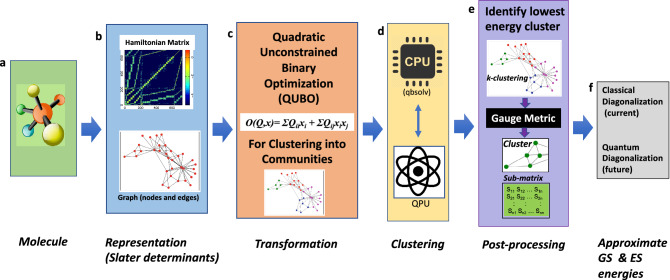
Conceptual workflow for the reduction process. (**a**) Cartesian co-ordinates for atoms, atomic numbers and a chosen basis set define a molecule. (**b**) An electronic Hamiltonian matrix is constructed in the basis of Slater Determinants (SDs) of spin-orbitals by using a classical quantum chemistry code. This matrix is further represented as a graph adjacency matrix where each SD is a node. (**c**) The graph is transformed to a Quadratic  Unconstrained Binary Optimization (QUBO) form of *Community Detection*. (**d**) The QUBO is run using quantum-classical *qbsolv* + D-Wave quantum annealer. (**e**) The resulting clusters/communities as sub-matrices are candidates for the best low energy solution. The gauge metric is used to identify the lowest energy cluster. (**f**) Approximate ground state and excited state energies are calculated using diagonalization.

The conceptual workflow for this process is shown in Fig. 1. Starting from the Cartesian coordinates for atoms, atomic numbers and a chosen basis set defining a molecule (Fig. 1a), an electronic Hamiltonian matrix is constructed in the basis of SDs of spin-orbitals by using one of the classical quantum chemistry codes such as Psi4^30^. This matrix is represented as a graph (Fig. 1b) and is transformed into a QUBO form of *Community Detection* (Fig. 1c) to be solved on the D-Wave Quantum Annealer (Fig. 1d). Due to the matrix/graph size, a quantum-classical approach using *qbsolv* and the D-Wave is required. The resulting clusters or communities are then candidates for the best low-energy solution. The one containing the HF SD or identified from the gauge metric in the post-processing step (Fig. 1e) is the lowest energy cluster. The approximate ground state and excited state energies are calculated by diagonalization on a classical computer (Fig. 1f).

## Results

### The reduction process

The reduction approach proceeds as follows. A molecular Hamiltonian $${{\hat{H}}}$$ matrix in the basis of SDs serves as input. The *k-clustering* sets of the Hamiltonian matrix are explored starting with $$k=2$$. *Quantum Community Detection* performed using the D-Wave quantum annealer determines the *k* communities or clusters. Each of the *k* clusters translates into a sub-matrix of SDs. The one containing the HF SD (or identified by the gauge metric) is the cluster of interest out of the *k* clusters and yields the lowest energy, $$E_{CL}$$, when diagonalized classically. The results obtained by this procedure fall between the HF ($$E_{HF}$$) and FCI energy of the full matrix ($$E_{FCI}$$), with a tendency to be closer to $$E_{FCI}$$. The accuracy of the result can be measured as the difference with respect to $$E_{FCI}$$, (energy difference $$= E_{CL}-E_{FCI})$$. This can be repeated for *3-*, *4-*, *5-clustering* and more as needed. Each *k-clustering* is independent of the others. One does not need to go through the *k-clusterings* in order, but it is natural to do so. Based on knowledge about a molecule, one may even choose to start from a large *k* and work backwards. The cluster with the lowest energy difference over all *k-clustering* sets is the best result from an accuracy perspective. This sub-matrix is the reduced matrix, constituting an approximate representation of the full Hamiltonian. The goal of this procedure is to discover a reduced matrix with an energy difference within chemical accuracy. The focus can be on accuracy or size. One should expect the larger the size, the higher the accuracy. Multiple clusters with energy differences within chemical accuracy may be found. Any of these is sufficient for ground state energy calculations. We note here that the reduction method is not limited to the energy and could potentially be extended to compute the atomic forces, for geometry optimization or molecular dynamics.

### Illustrating the method with H_2_O

**Figure 2:**
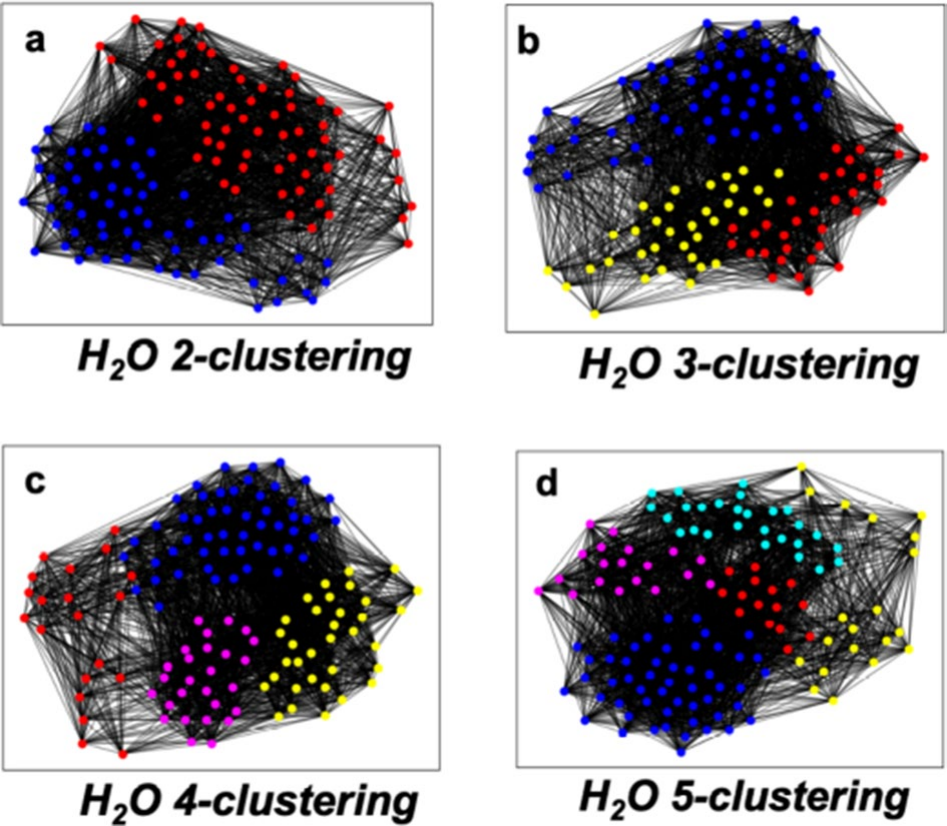
Showing H_2_O *2-*, *3-*, *4-*, and *5-clustering* from *Quantum Community Detection* on the D-Wave Quantum Annealer. For all the *k-clusterings*, the cluster or community of nodes with the lowest energy is shown in blue. (**a**) The *2-clustering* produced communities of size 68 (red) and 65 (blue). (**b**) The *3-clustering* is shown with communities of size 34 (red), 65 (blue), and 34 (yellow). The *2-* and *3-clustering* share the same low energy cluster of 65. (**c**) The *4-clustering* produced communities of size 20 (red), 52 (blue), 34 (yellow), and 27 (magenta). (**d**) The *5-clustering* resulted in communities of size 15 (red), 52 (blue), 20 (yellow), 19 (magenta), and 27 (cyan). The *4-* and *5-clustering* share the same low energy cluster of 52. The clustering pictures were generated using NetworkX 2.5^31^ (https://networkx.org) and Matplotlib 3.3.3^32^ (https://matplotlib.org).

The Hamiltonian FCI matrix of the H_2_O molecule in the sto-3g minimal basis set is a 133 $\times$ 133 sparse Hermitian (i.e., real symmetric) matrix after imposing the unitary groups U(1) and SU(2) (spin and particle conservation) and $\hbox {C}_{2V}$ point group symmetries. The sparse matrix can be represented as a weighted graph of 133 nodes with 3032 edges. Each off-diagonal matrix element is considered an edge weight. Running *Quantum Community Detection* for *2-clustering* produces the communities shown in Fig. 2a. The 65 nodes of the blue community represent the SD indices of the reduced matrix at this level with the lowest energy. The energy difference for this cluster is 0.05 kcal/mol for the lowest eigenvalue (ground state energy), which is achieved by almost a 2-fold decrease in matrix size. The *3-clustering* shown in Fig. 2b produces two other communities and the blue community with the same ground state energy. A further reduction occurs with *4-clustering* as shown in Fig. 2c. Classical diagonalization of this blue community of 52 SDs produces a ground state energy value with an acceptable energy difference of 0.68 kcal/mol. No further reduction is seen with *5-clustering*, as shown in Fig. 2d. A significant 49% and 39% of the original molecule Hamiltonian matrix is retained with the 65 and 52 SD communities.

An excited state of a molecule is any electronic quantum state of the system that has a higher energy than the ground state. Table 1 also shows the energy differences for the H_2_O ground state and next five excited states for the reduced matrices based on the communities of size 65 (CL65) and 52 (CL52). Refer to the Supplementary Information for additional data. Chemically accurate energies for five excited states are shown for CL65 and only the first three for CL52. This indicates that the lower accuracy CL52 does not contain all the SDs necessary for higher excited state calculations.

**Figure 3:**
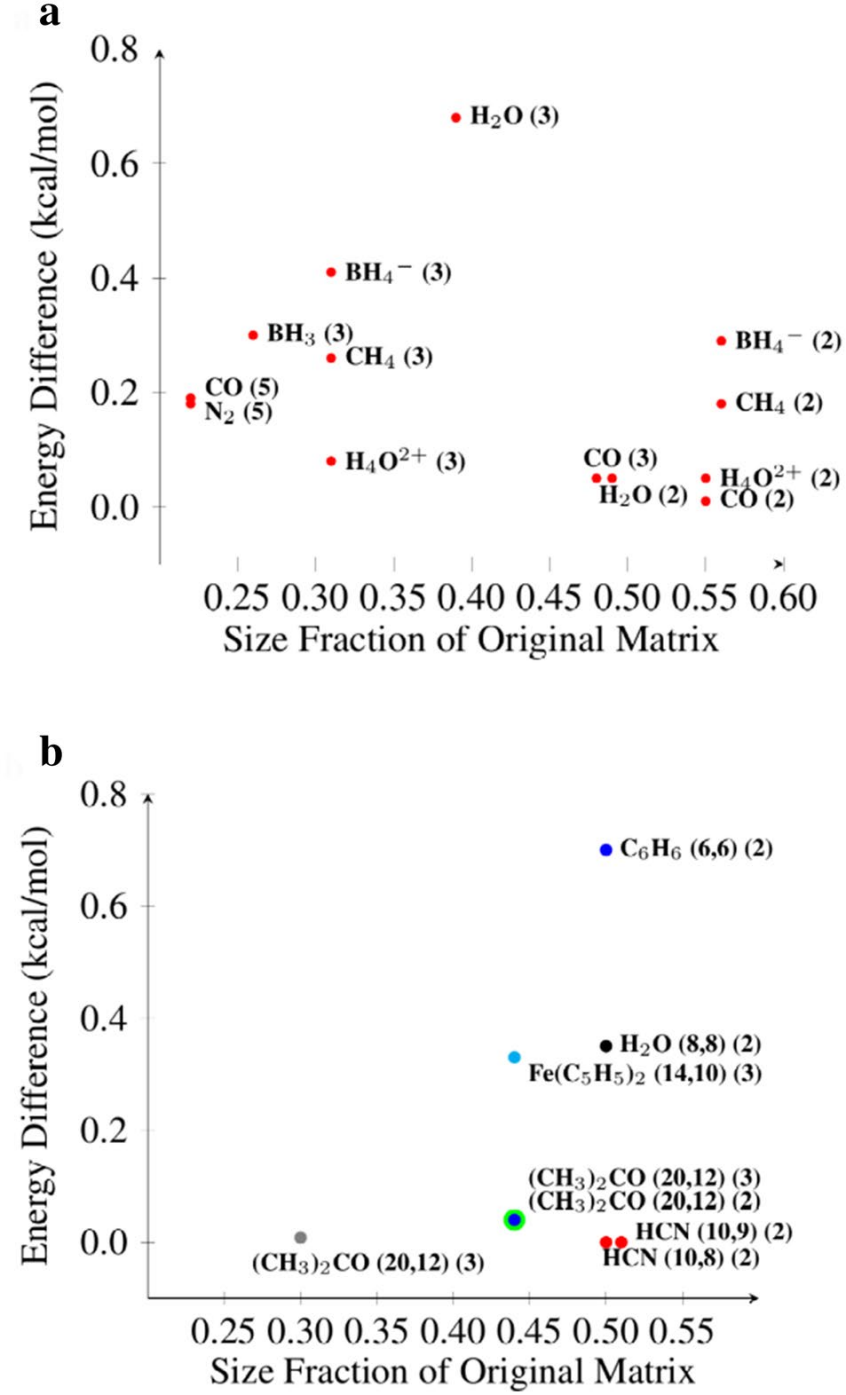
Comparison of energy difference and size reduction for different molecule clusters within chemical accuracy. (**a**) Molecule clusters using FCI and the sto-3g minimal basis set are shown. Those in the lower right corner from a *k-clustering* with $k=2$, shown as “(2)”, are the best results from an accuracy perspective. The others have smaller size and less accuracy. (**b**) Molecule clusters using CASSCF and different basis sets are shown. These are all from a *2-* or *3-clustering*. In this case the accuracy is independent of size. The basis set used for each molecule is noted by color: sto-3g is red, cc-PVQZ is blue, def2-svp is gray, def2-TZVP is cyan, cc-PVDZ is black, and 6-31G$\star$ is green.

**Table 1:** Approximate ground and excited states energies for H_2_O in the sto-3g basis.

Excited state	$E_{CL65}-E_{FCI}$ (kcal/mol)	$E_{CL52}-E_{FCI}$ (kcal/mol)
0	0.05	0.68
1	0.06	0.19
2	0.09	0.49
3	0.02	0.51
4	0.04	33.15
5	0.13	17.07

### Molecules using FCI and the sto-3g minimal basis set

Quantum chemists are predominantly interested in ground state energies. Molecular systems used to create FCI matrices in the sto-3g minimal basis set for further evaluation of the performance of *Quantum Community Detection* are shown in Fig. 3a. Refer to the Supplementary Information for additional data. H_2_O, CO, CH_4_, BH_4_^-^, and H_4_O^2+^ show a similar pattern for the *k-clustering* that gave the best low energy result. The *2-clustering* produced a relevant cluster that gave good initial results (within chemical accuracy, i.e. < 1 kcal/mol), followed by a *3-* or *4-clustering* cluster that reduced the matrix size further at slightly lower accuracy. BH_3_ and N_2_ show a different pattern. A *k-clustering* with $k>$ 2 produced a cluster with greater reduction in size and still within chemical accuracy. Figure 3a shows the same molecules in comparison based on the size fraction of the original vs. the energy difference relative to FCI. Only the reduced molecule clusters resulting in energies within chemical accuracy are shown. Those from *2-clustering* produced the most accurate results with ~50% reduction in size. Those from clusterings where $k>$ 2 produced clusters of smaller size, though still within chemical accuracy.

### Molecules using the CASSCF method

The complete active space self-consistent field method (CASSCF) is a multi-configurational SCF method^6–8^. The active space consists of the electrons and orbitals that are necessary for a reliable description of the electronic structure of a molecule. CASSCF can be thought of as a truncation of FCI, which is based on a full active space within which all orbitals are considered. The total number of orbitals depends directly on the number and type of atoms in the system. Given a number of electrons (e) distributed across a number of orbitals (o), a Hamiltonian consisting of a linear combination of SDs or configuration state functions (CSFs) is obtained. Careful design of the active space requires chemical intuition based on the molecule of interest^33,34^. This method is useful when HF and DFT are not adequate (frequently the case of static electronic correlations), and FCI is computationally intractable. *Quantum Community Detection* could potentially be used for further optimal truncation of the initially chosen active space.

Molecules used to create the CASSCF matrices in different basis sets are shown in Fig. 3b. For HCN (hydrogen cyanide) and (CH_3_)_2_CO (acetone), the choice of active space was standard, based on the highest occupied molecular orbital as a reference point. For C_6_H_6_ (benzene), Fe(C_5_H_5_)_2_ (ferrocene) and C_8_H_10_N_4_O_2_ (caffeine), the choice of active space was chemically inspired (see Supplementary Information). Acetone with different basis sets and HCN with different active spaces show the best accuracy. Clusters with less accuracy are seen for C_6_H_6_, Fe(C_5_H_5_)_2_, and H_2_O. The sizes, energies, and energy differences along with additional data are shown in the Supplementary Information. For most of these cases, chemical accuracy was achieved with the *2-* or *3-clustering* sets, as shown in Fig. 3b. Additionally, two molecules, HCN (10,9) using the 6-31G* basis set and caffeine using the sto-3g basis set resulted in low energy clusters greater than chemical accuracy (see Supplementary Information). In the case of caffeine, expert chemical knowledge was used in choosing the original active space of 12 electrons across 9 orbitals and left no room for improvement. The gauge metric indicated that this could not be reduced further while retaining chemical accuracy (see Supplementary Information). This was further confirmed by *Quantum Community Detection* for *2-clustering* which led to an energy difference of 24.36 kcal/mol.

**Figure 4:**
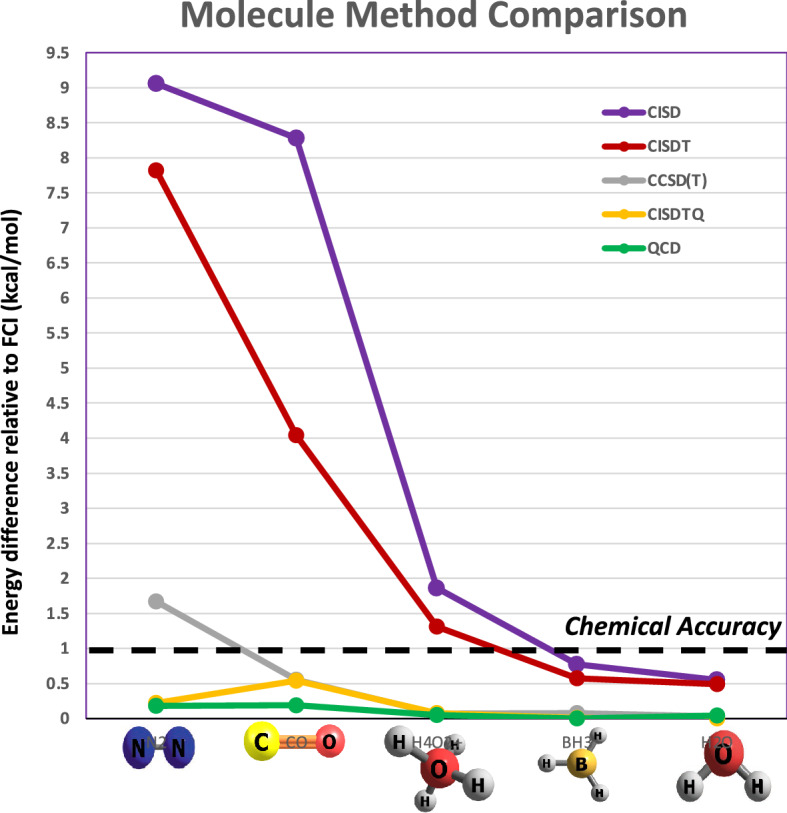
Comparison of *Quantum Community Detection* with current methods. Results for CISD, CISDT, CCSD(T), CISDTQ, and our *Quantum Community Detection* (QCD) relative to FCI are shown for five molecules, N_2_, CO, H_4_O^2+^, BH_3_, and H_2_O. CISD (violet), CISDT (red) and CCSD(T) (gray) produced energy differences greater than chemical accuracy for one or more molecules. For these five molecules we can see that *Quantum Community Detection* produced results that are competitive with current methods, similar to CISDTQ and the gold standard, CCSD(T).

### Comparison with other methods

In Fig. 4, we compare *Quantum Community Detection* with commonly used current methods, CISD, CISDT, CCSD(T), and CISDTQ, all relative to FCI. The hierarchy of approximations consists of HF $\Rightarrow$ CISD $\Rightarrow$ CISDT $\Rightarrow$ CCSD(T) $\simeq$ CISDTQ $\Rightarrow$ FCI. The inclusion of higher excitations leads to a larger expansion and produces an answer which is typically closer to the exact solution. The higher configuration interaction methods are more accurate, but at the expense of an increased problem size which scales steeply ($N^6$ to $N^{10}$). Figure 4 shows the energy differences for the five methods across the five molecules (N_2_, CO, H_4_O^2+^, BH_3_, and H_2_O) analyzed here. In this case, CISD, CISDT, and CCSD(T) produced results greater than chemical accuracy for one or more molecules. The size produced by *Quantum Community Detection* compared to CISDTQ and CCSD(T) is smaller or similar (see Supplementary Information). In general, for this small set of molecules, we can see that *Quantum Community Detection* produces results that are competitive with current methods, in line with CISDTQ and the gold standard method, CCSD(T).

**Figure 5:**
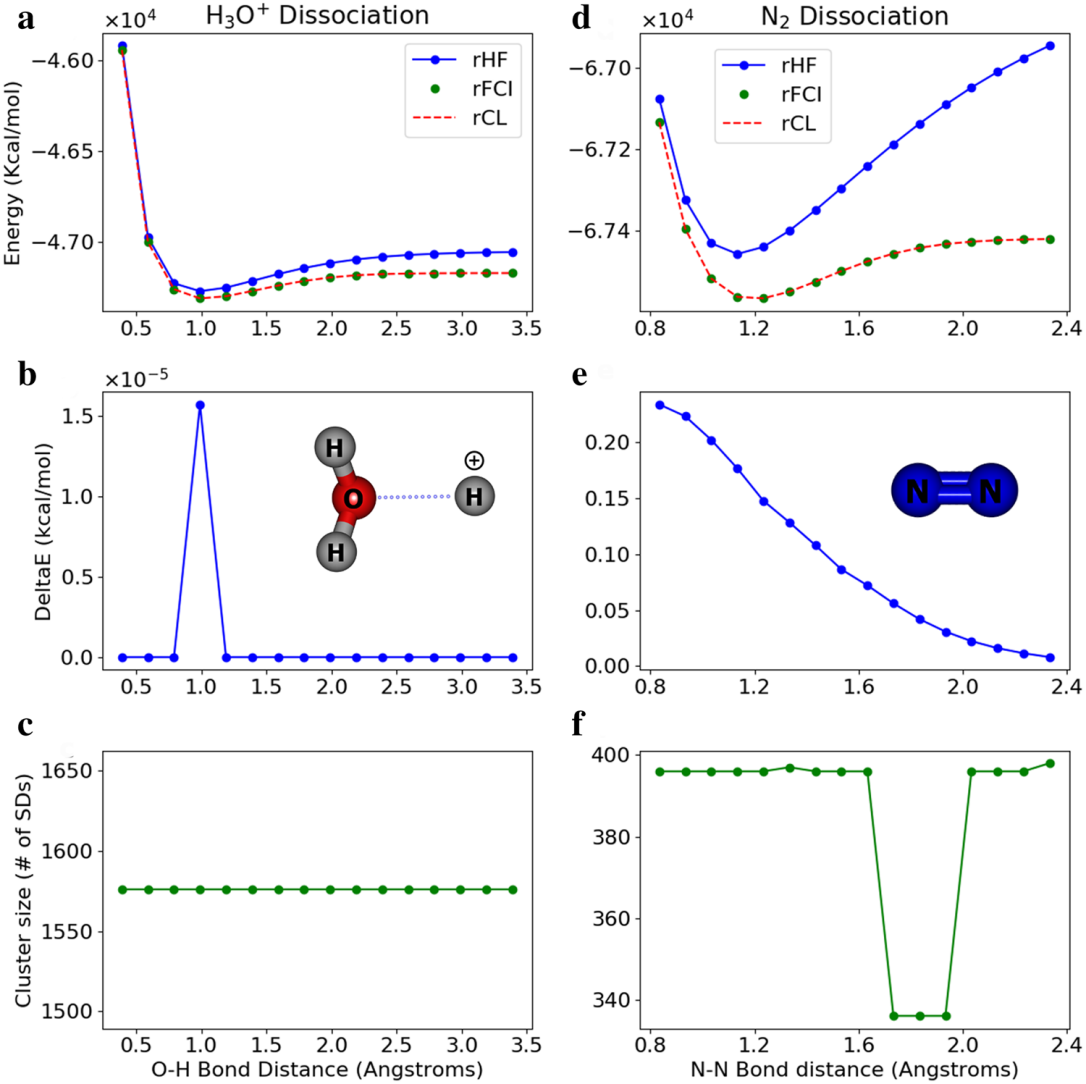
Bond dissociation for H_3_O^+^ and N_2_. (**a**) Showing H_3_O^+^ bond dissociation using the reduced sub-matrices discovered using *Quantum Community Detection*. The lowest energy bond distance is 0.99 Å. The FCI and cluster (CL) energies across all distances are nearly the same. (**b**) Most of the energy differences are within $10^{-10}$ kcal/mol across all O–H bond distances, though the lowest energy point is within $10^{-05}$ kcal/mol. (**c**) The reduced sub-matrix size for all bond distances is 1576 (from low energy clusters). This is a 50% reduction from the original matrices of size 3136 SDs. (**d**) Showing potential energy curve for N_2_ bond dissociation. (**e**) N_2_ energy differences decrease with larger bond distance. (**f**) Reduced sub-matrix sizes for N_2_ vary from 363 to 397 SDs out of 1824 across available bond distances.

### H_3_O^+^ bond dissociation

A bond dissociation energy experiment was performed for H_3_O^+^ for O–H bond distances of 0.39–3.39 Å by enforcing $\hbox {C}_{2V}$ molecular symmetry. We simulated a constrained potential energy surface of an oxonium ion (H_3_O^+^) in the sto-3g basis (FCI method), as shown in Fig. 5a. Specifically, the constrained parameter was chosen as an O–H bond. Starting from the optimized geometry of H_3_O^+^, the latter was stretched/shortened by using a step of 0.2 Å in a constrained scan optimization (at HF level). The overall process can be viewed as a protonation of H_2_O. The optimized geometries were used to generate FCI matrices of size 3136. The *Quantum Community Detection* method resulted in reduced matrices of size 1576 for all geometries (see Fig. 5c), a 50% reduction. Their diagonalization provided approximate ground state energies within 1.57 $\times 10^{-5}$ kcal/mol relative to the original FCI matrices as seen in Fig. 5b. Refer to the Supplementary Information for data on all the clusterings performed.

### N_2_ bond dissociation

A bond dissociation energy experiment was performed for N_2_ for distances of 0.5–2.4 Å. The ground state for each N_2_ matrix was optimized using the restricted Hartree–Fock (RHF) method and the sto-3g basis set. The Hamiltonian matrices were generated using Psi4^30^ along with the rHF and rFCI energies. Low energy communities were discovered for each bond distance using our *Quantum Community Detection* approach producing energies well within chemical accuracy as seen in Fig. 5d–f and the Supplementary Information. Interestingly, the $E_{rCL}-E_{rFCI}$ values decrease with increasing bond distance. The reduced sub-matrices are all similar in size ranging from 363 to 397 SDs (see Fig. 5f) as compared to the original 1824 SDs, up to a 4-fold reduction in size. It is a well-known fact that RHF cannot qualitatively describe the electronic structure in a molecule undergoing homolytic bond breaking. N_2_ has been widely used as a test molecule for many multi-reference approaches^35^. However, the bond-dissociation curve is quantitatively captured by the *Quantum Community Detection* method shown in Fig. 5d, which indicates that the method successfully selects the reduced sub-matrices that include all the important SDs to recover the correct electronic structure along the dissociation curve.

### Cluster analysis based on energy and connectivity

The best low energy cluster for a molecule from *Quantum Community Detection* depends on the energy of the individual SDs in a cluster and the connectivity between them. Community detection determines the clusters by relying on the off-diagonal values of the Hamiltonian matrix as edge weights between SDs as the connectivity contribution.

We observe that the energy difference depends on the cluster, and it is not necessarily a monotonic function of the number of clusters within a *k-clustering*. Because of this, there is an optimal *k* after which, if *k* is further increased, important elements from the Hamiltonian matrix are lost, and the energy difference is further increased.

A metric to “gauge” the energy difference for the corresponding sub-matrix of each cluster of a particular *k-clustering* can be calculated involving SD energies (diagonal elements of the Hamiltonian matrix) and the weights between SDs (off-diagonal elements of the Hamiltonian matrix). Notably, the former quantity is related to the energies of spin-orbitals composing a given SD, which historically served as a criterion for selecting active spaces for truncated methods. Therefore, an analysis of the diagonal elements of the Hamiltonian may be useful to rationalize the *Quantum Community Detection* truncation. The cluster with the lowest metric will also result in the lowest energy and smallest energy difference for this set. This reduces the diagonalization calculations down to one for each *k-clustering*. The energy difference can be gauged with the approximations typically used in quantum chemistry, such as perturbation theory or energy re-normalization^36^.

**Figure 6:**
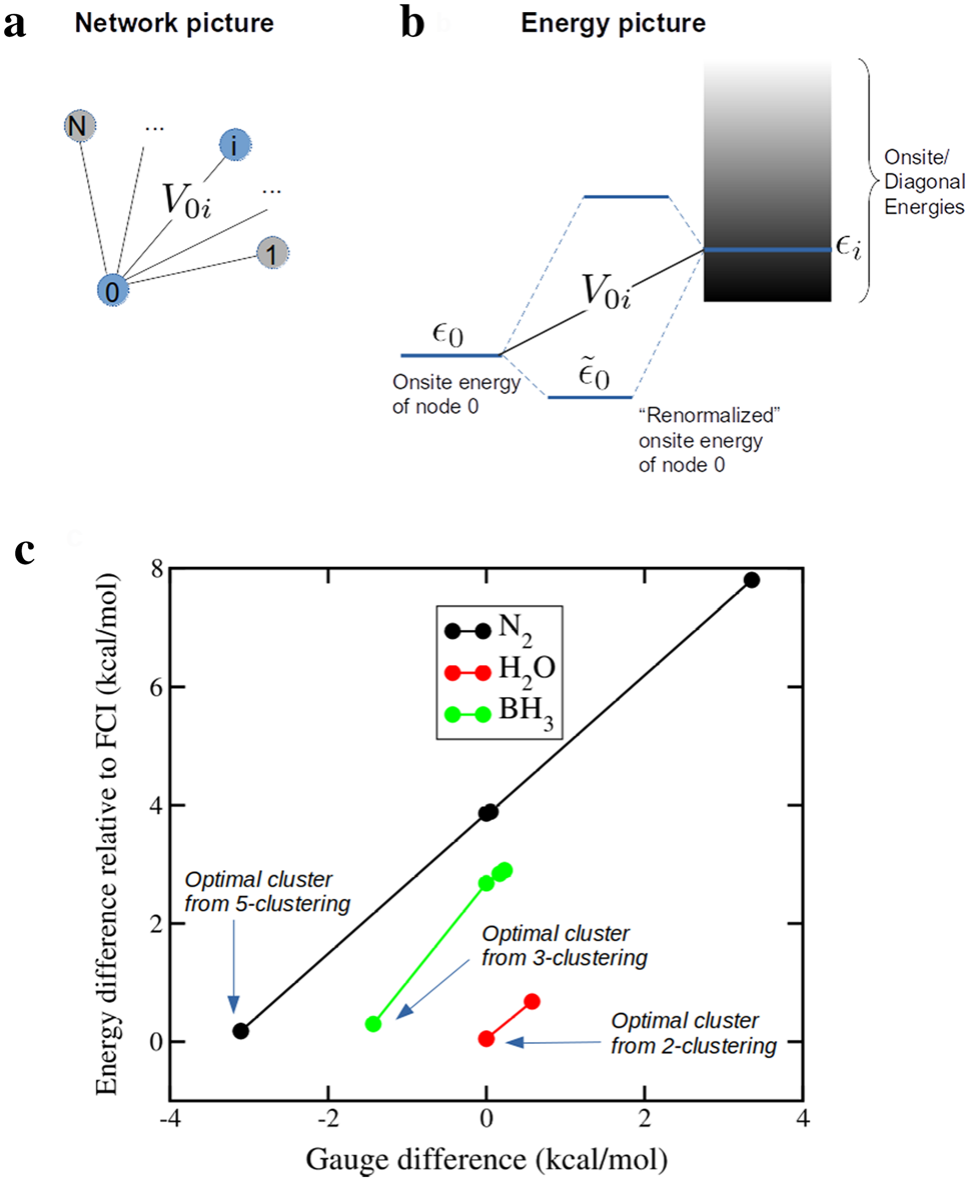
Gauge metric description. (**a**) Network representation of a cluster. (**b**) Scheme of how the energy of node 0 (diagonal element $\epsilon _0$) gets re-normalized (corrected) to ${\tilde{\epsilon }}$ due to the interaction with the other nodes (SDs) via the coupling elements or weights $V_{0i}$. (**c**) Gauge and energy differences are shown for H_2_O, BH_3_, and N_2_. The lowest gauge metric differences and corresponding energy differences denote the most accurate cluster. Pair-wise differences in the gauge metrics and the corresponding energy differences have a linear relationship as shown. The most accurate cluster across all *k-clusterings* is noted.

Following this same idea, we correct the energy by considering the interaction of multiple states. In Fig. 6a we can see a network representation of an SD (node) interacting with many others within a network representation and within an energy representation. In Fig. 6b, the energy representation, we show how the energy of node 0 (diagonal element $\epsilon _{0}$) gets re-normalized (corrected) to ${\tilde{\epsilon }}_0$ due to the interaction with another node via the coupling elements or weights $V_{0i}$. The gauge metric *f* calculated across all SDs in a cluster is shown in Eq. (3). A value of $\delta$ is added to the denominator to avoid a division by 0. This parameter is typically known as “phase shift” or “denominator shift” within the context of many-body methods^37,38^. The value of $\delta$ will need to be determined for every particular problem. For the cases analyzed in this manuscript, a value of $\delta$ = 1.0 not only shows monotonicity with the energy difference, it is also linear. Refer to the Supplementary Information for a detailed derivation.

3 $$\begin{aligned} f = \mathrm {min}_{i} \left( \epsilon _{ii} - \sum _k\frac{V^2_{ik}}{|\epsilon _{ii} - \epsilon _{kk}| + \delta }\right) \end{aligned}$$

The metric *f* can be used to determine the relevant cluster of a *k-clustering* without having to compute *k* diagonalizations. Although we currently have a partial understanding of why there is no clear decreasing monotonicity between *k* and the accuracy of the selected cluster; the explanation of the differences in accuracy between clusters are fully contained in the factors of this gauge metric *f*. This metric is derived and analyzed further in the Supplementary Information.

Looking at the pair-wise differences in the metric *f* values of the best clusters across multiple *k-clusterings*, we see that the lowest difference determines the cluster with the best accuracy for a given molecule. This relationship between the metric *f* differences and the energy differences is monotonic and linear as shown in Fig. 6c for H_2_O, N_2_, and BH_3_. The most accurate cluster for H_2_O was discovered from the *2-clustering* with a small energy difference of 0.05 kcal/mol. This remained the same for *3-clustering* resulting in a metric *f* difference of 0.0. For N_2_, the smallest energy difference of 0.18 kcal/mol and a low metric *f* difference between the *4-* and *5-clustering* confirm that the most accurate cluster comes from the *5-clustering*. Similarly, for BH_3_, the smallest energy difference of 0.30 kcal/mol and lowest metric *f* difference between the *3-* and *2-clustering* indicate that the most accurate cluster is from the *3-clustering*. These results are shown in the Supplementary Information. The most accurate cluster includes low energy SDs (the lower the better), which are close in energy, with high weights between the SDs (as nodes). The gauge thus provides a quick test if the inclusion of low energy SDs is sufficient for a given *k*. The gauge can also be used to indicate whether a low energy cluster will result in chemical accuracy or not (see Supplementary Information).

**Figure 7:**
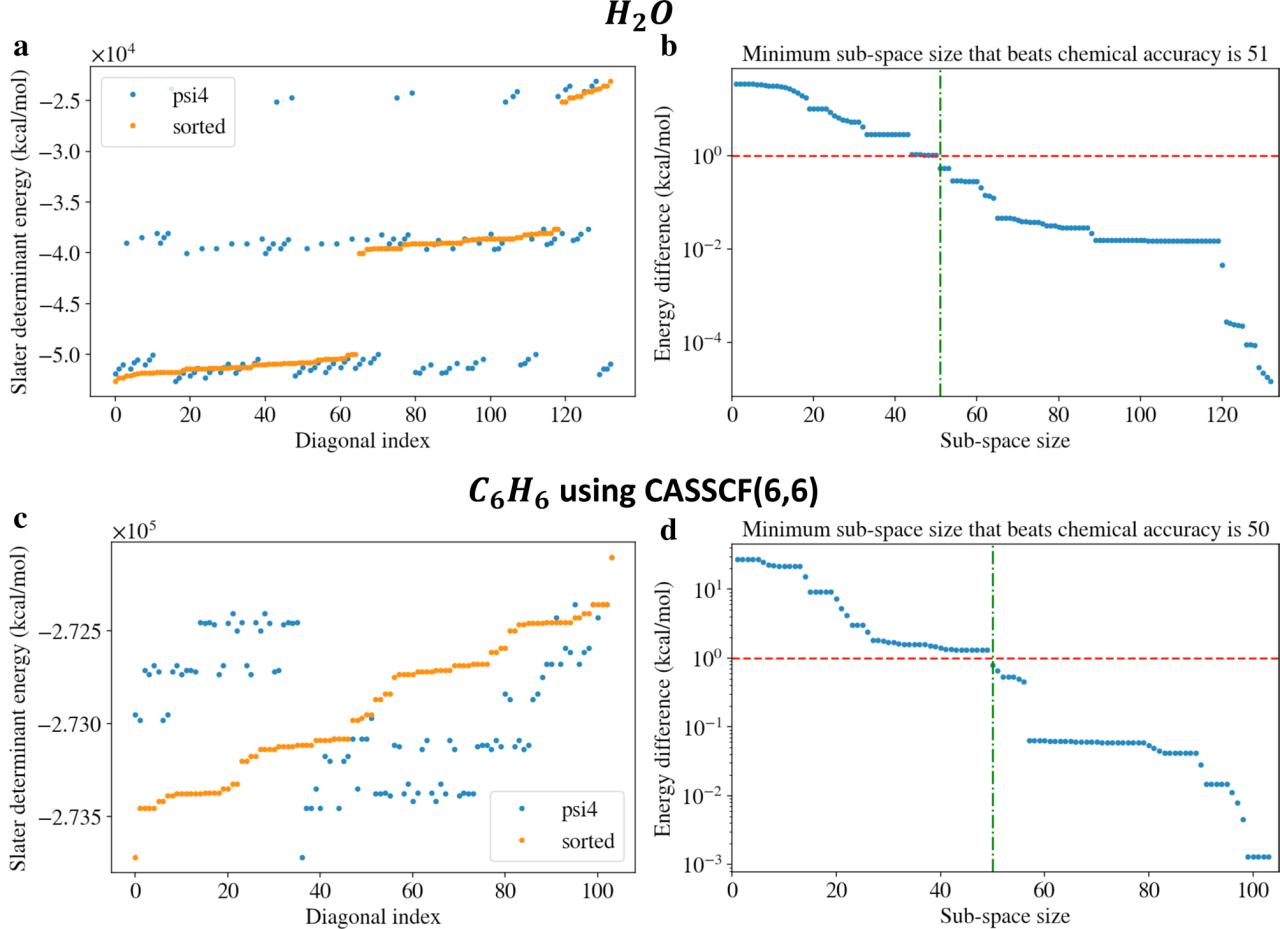
The sorted energy approach is demonstrated for H_2_O and C_6_H_6_. (**a**) The SDs for H_2_O in sorted order are shown displaying distinct energy levels. (**b**) The minimum number of SDs required to be within chemical accuracy, 51, is shown. (**c**) The sorted SDs for C_6_H_6_ are shown, somewhat separated in energy. (**d**) The minimum number of SDs within chemical accuracy, 50, is shown.

Could better clusters be formed from the lowest energy SDs only? Arranging the SDs in sorted order and making “brute force” diagonalization calculations for clusters of increasing size, showed that this approach works for some cases. In Fig. 7, we show the results for H_2_O and C_6_H_6_ from a sorted energy perspective. Sorting the H_2_O SDs from low to high energy clearly shows three energy levels (see Fig. 7a). The minimum number of SDs required to be within chemical accuracy (just less than 1.0 kcal/mol) is 51 (see Fig. 7b). In comparison, *Quantum Community Detection* results in 65 SDs for high accuracy and 52 SDs for lower accuracy. Considering C_6_H_6_, the sorted SDs are somewhat separated in energy as seen in Fig. 7c. The minimum number of SDs to be within chemical accuracy is 50, just below 1.0 kcal/mol as seen in Fig. 7d. *Quantum Community Detection* results in 52 SDs, with an energy delta of 0.70 kcal/mol and 28 SDs for an energy delta of 0.72 kcal/mol. Within a *k-clustering*, the majority of the SDs included in the lowest energy cluster are of low energy. However, a more complex interplay between different SDs could further lower the energy as can be deduced from the factors of the metric *f*.

## Discussion

The solution of the Schrödinger equation lies at the heart of computational chemistry and is usually accomplished by numerical diagonalization of the Hamiltonian matrix to obtain energies of the ground and excited states. Over decades, exponential hardness of the original problem was eased in practice by various many-body approximations allowing for reduction of the Hamiltonian size. Availability of quantum computing hardware provides new and unconventional means to solve traditionally difficult numerical problems. While the majority of quantum efforts have been devoted to turning exponential-to-polynomial scaling of numerical expenses, automatic reduction of the Hamiltonian size on the quantum hardware remains an important algorithmic development with practical implications. Here we have introduced a new approach for reducing the molecular Hamiltonian matrix for electronic structure calculations using *Quantum Community Detection* running on the D-Wave quantum annealer. The algorithm automatically samples the Hamiltonian space and identifies clusters allowing for accurate approximation of the lowest eigenvalue of the Hamiltonian through diagonalization that corresponds to the ground state energy of the molecule.

We have applied our *Quantum Community Detection* method to a variety of different molecular systems of different sizes and chemical properties. For several cases, the resulting clusters produced ground state energies within chemical accuracy ($\le$ 1 kcal/mol) providing size reductions of 50% or more, and serve as good approximations to the original larger problems (i.e. calculations at full configuration interaction level). We note here that the approach is not variational, however, and does not guarantee a reduction within chemical accuracy. Though the gauge metric can be used to indicate whether reduction is possible and if the reduced sub-matrix will be within chemical accuracy.

Beyond exploring chemical diversity, we have also demonstrated application to two chemical reaction/conformational dynamics examples. Bond dissociation of H_3_O^+^ achieved a ~ 50% reduction in matrix size while maintaining chemical accuracy. While N_2_ bond dissociation required only ~ 25% of the original matrix size, also within chemical accuracy.

We further have shown connections to the traditional approaches of Hamiltonian reduction via selecting molecular orbitals that provide low-energy Slater Determinants (SD). Our analysis shows that both the energies of the SDs and the connectivity between them are important in determining the best approximate representation of a molecule within chemical accuracy for electronic structure calculations. The high accuracy clusters overall include mostly low energy SDs (the lower the better), which are close in energy. However, they may also include high energy SDs as well and the connectivity or weights between the SDs (as nodes) is typically high. The *Quantum Community Detection* approach was generally able to reduce the size of the molecular Hamiltonian without chemical knowledge or intuition. For the case study of five molecules, the algorithm resulted in an efficient reduction of Hamiltonian space toward reaching chemical accuracy, which is competitive with the current traditional approaches achieving reduction by limiting configuration interaction expansion (i.e. CISD, CISDT, CCSD(T), and CISDTQ levels).

Similar to selected configuration interaction (sCI)^39,40^, the method presented in this paper is able to prune the space of all directly interacting states in the Hamiltonian matrix as a graph to account for the most important ones that are representative of the original. The initial FCI matrix still needs to be constructed and the resulting reduced sub-matrix still needs to be diagonalized. In our proof-of-principle paper, they were required to gain an understanding of the results and provide ground truth comparisons. Working with larger molecular systems with potentially larger FCI matrices we will not have this luxury and will need to alleviate this by closer integration with the FCI matrix construction producing a reduced sub-matrix as part of the process. Techniques such as sCI, incremental FCI construction^41^, and Monte Carlo CI methods^42^ that operate on batches may be helpful here. Diagonalization can be avoided by using perturbation-based methods. The *Quantum Community Detection* reduction method could be used as part of sCI or as a post-processing step to further reduce the matrix. It could also be used as a pre-conditioner combined with other methods such as density matrix renormalization group (DMRG)^43^, where a reduced Hamiltonian sub-matrix could improve the process. These ideas will be explored in future studies.

*Quantum Community Detection* formulated for the D-Wave quantum annealer is a promising algorithmic development making use of emerging quantum hardware toward solving important quantum chemical problems. This study reports proof-of-principle concept, while practical utility of this algorithm requires further investigation. This motivates further studies targeting exploration of deeper insights into the relevance of quantum communities and energetics of SDs and molecular orbitals. Analysis of the molecular orbitals contributing to the communities will allow for uncertainty quantification and construction of algorithms gradually expanding an active space as well as reduction.

## Methods

### Molecular Hamiltonian matrix preparation

All molecular geometries were first optimized at the restricted HF level (gas) with Gaussian 09 (E01) code^44^. The FCI and CASSCF matrices were generated using the in-house modified Psi4 code^30^ with the optimized Cartesian coordinates of atoms as input data. The matrices were generated imposing the unitary groups U(1) and SU(2) (spin and particle conservation) and point group symmetries and contain nonzero matrix elements only. If optimized by Gaussian 09, the molecule belongs to a non-Abelian point group, Psi4 automatically lowers the symmetry to one of its subgroups. The energy threshold of $10^{-8}$ $E_h$ for diagonal and of $10^{-10}$ $E_h$ for non-diagonal elements was set up. The nuclear repulsion (NR) term was added manually for clustered matrices.

### D-Wave 2000Q setup

The D-Wave 2000Q Ising resource at Los Alamos National Laboratory was used for this project. The solver used was *DW_2000Q_LANL* with 2032 active qubits (out of 2048) and 5924 active couplers (out of 6016). Default parameter settings were used for all runs (with an annealing time of 20 microseconds). All runs were quantum-classical using D-Wave’s *qbsolv* with a sub-qubo size of 65 for the D-Wave quantum annealer.

## Supplementary information

Supplementary Information.

## Data Availability

The data that support the findings of this study are available from the corresponding author upon reasonable request.
