# Supplementary material for: Reduction of the molecular hamiltonian matrix using quantum community detection
Source: Sci Rep. 2021 Feb 18;11:4099. doi: 10.1038/s41598-021-83561-x (PMC7892829; doi:10.1038/s41598-021-83561-x)
Supplement: Supplementary file 1 — Supplementary Information. [file 41598_2021_83561_MOESM1_ESM.pdf]

# Supplementary Information for Reduction of the Molecular Hamiltonian Matrix using Quantum Community Detection

Susan M. Mniszewski[1] *, Pavel A. Dub[2], Sergei Tretiak[3], Petr M. Anisimov[4], Yu Zhang[3], & Christian F. A. Negre[3]

[1] *Computer, Computational and Statistical Sciences Division, Los Alamos National Laboratory, Los Alamos, NM*
[2] *Chemistry Division, Los Alamos National Laboratory, Los Alamos, NM*
[3] *Theoretical Division, Los Alamos National Laboratory, Los Alamos, NM*
[4] *Accelerator Operations and Technology Division, Los Alamos National Laboratory, Los Alamos, NM*

**Supplementary Results**

Expanded tables are shown with more details including molecule point groups, FCI energies, and cluster energies. Additional *k-clustering* results are also included.

**Molecules using FCI and the sto-3g minimal basis set.** Molecules created using FCI and the sto-3g minimal basis set are shown in Table S 1. Table S 2 shows the energy for the $H_2O$ ground state and next five excited states for the reduced matrices based on the communities of size 65 (CL65) and 52 (CL52). Chemically accurate energies for five excited states are shown for CL65 and only the first three for CL52.

**Molecules using the CASSCF method.** Table S 4 shows extended data for molecules using the CASSCF method in addition to what is shown in the main manuscript. The choice of active space for the benzene, caffeine, and ferrocene molecules is shown in Figure S 1.

**Molecules using other truncation methods and extended basis sets.** Table S 3 shows molecules with different truncation methods and extended basis sets. Results with energy differences within chemical accuracy are highlighted. Molecule matrices of SDs created using the CIS truncation (CI + single excitations) are chemically known to have the first determinant in the matrix (the Hartree-Fock determinant) result as the lowest eigenvalue. This means there is no correlation between the first SD and any of the other SDs. This community of 1 SD is discovered at $k = 5$ for the 2 benzene examples in Table S 3. The energy difference remains the same for $k = 2$ to $k = 5$ since this 1 SD is present in all.

Table S 1: Molecules using the FCI method and the sto-3g minimal basis set.

| Molecule (Point Group) | Size | $E_{FCI}$ (Hartree) | $E_{CL}$ (Hartree) | k | Reduced Size | $E_{CL} - E_{FCI}$ (kcal/mol) |
|---|---|---|---|---|---|---|
| $H_2O$ | 133 | -75.02039100 | -75.02031690 | 2 | 65 | 0.05 |
| ($C_{2v}$) | | | -75.01930565 | 4 | 52 | 0.68 |
| CO | 3648 | -111.36801449 | -111.36799244 | 2 | 2013 | 0.01 |
| ($C_{\infty v} \Rightarrow C_{2v}$) | | | -111.36793125 | 2 | 1768 | 0.05 |
| | | | -111.36793125 | 3 | 1768 | 0.05 |
| | | | -111.36016556 | 4 | 1143 | 4.92 |
| | | | -111.36770868 | 5 | 792 | 0.19 |
| $CH_4$ | 4076 | -39.80512049 | -39.80484039 | 2 | 2284 | 0.18 |
| ($T_d \Rightarrow C_{2v}$) | | | -39.80469968 | 3 | 1284 | 0.26 |
| $BH_4^-$ | 4076 | -26.61685162 | -26.61639066 | 2 | 2284 | 0.29 |
| ($T_d \Rightarrow C_{2v}$) | | | -26.61620071 | 3 | 1284 | 0.41 |
| $H_4O^{2+}$ | 15876 | -75.36527671 | -75.36519709 | 2 | 8820 | 0.05 |
| ($C_1$) | | | -75.36514440 | 3 | 4900 | 0.08 |
| $BH_3$ | 1250 | -26.12145752 | -26.11717567 | 2 | 625 | 2.69 |
| ($C_{2v}$) | | | -26.12097251 | 3 | 321 | 0.30 |
| $N_2$ | 1824 | -107.66863149 | -107.66247136 | 2 | 1036 | 3.87 |
| ($D_{\infty h} \Rightarrow D_{2h}$) | | | -107.65620751 | 3 | 648 | 7.80 |
| | | | -107.66241960 | 4 | 544 | 3.89 |
| | | | -107.66835021 | 5 | 396 | 0.18 |
| | | | -107.66835021 | 6 | 396 | 0.18 |

Table S 2: Excited States for $H_2O$.

| Excited State | $E_{FCI}$ (Hartree) | $E_{CL65}$ (Hartree) | $E_{CL65} - E_{FCI}$ (kcal/mol) | $E_{CL52}$ (Hartree) | $E_{CL52} - E_{FCI}$ (kcal/mol) |
|---|---|---|---|---|---|
| 0 | -75.02039100 | -75.02031690 | 0.05 | -75.01930565 | 0.68 |
| 1 | -74.53743573 | -74.53734050 | 0.06 | -74.53713325 | 0.19 |
| 2 | -74.43197396 | -74.43183640 | 0.09 | -74.43118524 | 0.49 |
| 3 | -74.31751274 | -74.31748072 | 0.02 | -74.31670398 | 0.51 |
| 4 | -74.07950770 | -74.07943762 | 0.04 | -74.02668361 | 33.15 |
| 5 | -74.00260411 | -74.00238742 | 0.13 | -73.97540263 | 17.07 |

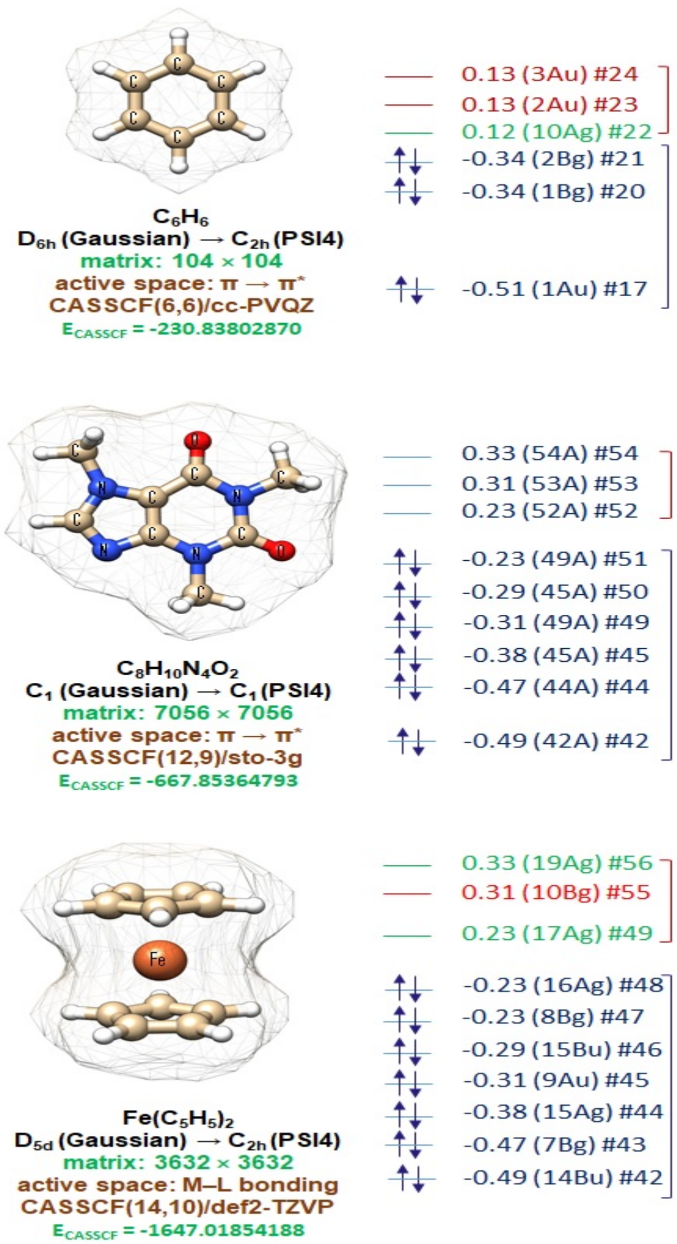


Figure S 1: Chemically-inspired choice of active space for CASSCF calculations of benzene (top), caffeine (middle) and ferrocene (bottom). For each numbered molecular orbital, energy (in Hartree) and irreducible representation is shown.

Table S 3: Molecules using CIS and extended basis sets.

| Molecule (Point Group) | Basis Set | Size | $E_{CL}$ (Hartree) | k | Reduced Size | $E_{CL} - E_{FCI}$ (kcal/mol) |
|---|---|---|---|---|---|---|
| $C_6H_6$ | 6-31G* | 591 | -230.70313698 | 2 | 191 | 4.99e-10 |
| Benzene | | | | 3 | 115 | 4.99e-10 |
| ($D_{6h}$) | | | | 4 | 96 | 4.99e-10 |
| | | | | 5 | 1 | 4.99e-10 |
| $C_6H_6$ | cc-PVDZ | 651 | -230.72234960 | 2 | 207 | 5.35e-10 |
| Benzene | | | | 3 | 104 | 5.35e-10 |
| ($D_{6h}$) | | | | 5 | 1 | 5.35e-10 |

**Comparison with other methods.** In addition to the figure in the main manuscript, Table S 5 and Table S 6 show *Quantum Community Detection* (QCD) compared to other methods, currently in practice, CISD, CISDT, CCSD(T), CISDTQ, and FCI for 5 molecules. Size and energy are shown for $N_2$, CO, $H_4O^{2+}$, $BH^3$, and $H^2O$. QCD is comparable in energy and size for these 5 molecules.

**$N_2$ bond dissociation.** A bond dissociation energy experiment was performed for $N_2$ for distances of 0.5 to 2.4 Å. The ground state for each $N_2$ matrix was optimized using the restricted Hartree-Fock (RHF) method and the sto-3g basis set. The Hamiltonian matrices were generated using Psi4 [1] along with the rHF and rFCI energies. Low energy communities were discovered for each bond distance using our *Quantum Community Detection* approach producing energies well within chemical accuracy as seen in the figures in the main manuscript and Table S 7. Interestingly, the $E_{fCL} - E_{rFCI}$ values decrease with increasing bond distance. The reduced sub-matrices are all similar in size ranging from 363 to 397 SDs (see Table S 7) as compared to the original 1824 SDs, up to a 25% reduction in size.

**$H_3O^+$ bond dissociation.** The Tables S 8, S 9, and S 10 show all results from all *k-clusterings* that appear in the figures in the main text and more. A bond dissociation energy experiment was performed for $H_3O^+$ for O-H bond distances of 0.39 to 3.39 Å. We simulated a constrained potential energy surface of an oxonium ion (or hydronium cation) ($H_3O^+$) in sto-3g basis (FCI method). Specifically, the constrained parameter was chosen as an O-H bond. Starting from the optimized geometry of $H_3O^+$, the latter was stretched/shortened by using a step of 0.2 Å in a constrained scan optimization (HF). The overall process can be viewed as a protonation of $H_2O$. The optimized geometries were used to generate FCI matrices of size 3136. The *Quantum Community Detection* method produced reduced matrices of size 1576 for all geometries, a 50% reduction.

Table S 4: Molecules using the CASSCF method.

| Molecule (Point Group) | Basis Set | Size | $E_{CL}$ (Hartree) | k | Reduced Size | $E_{CL} - E_{FCI}$ (kcal/mol) |
|---|---|---|---|---|---|---|
| HCN (10,8) Hydrogen Cyanide ($C_1$) | sto-3g | 1576 | -91.82114958 | 2 | 792 | 0.000013 |
| HCN (10,9) Hydrogen Cyanide ($C_1$) | sto-3g | 8036 | -91.84350714 | 2 | 4076 | 0.000054 |
| HCN (10,9) Hydrogen Cyanide ($C_1$) | 6-31G* | 4076 | -93.00896883 | 2 | 2110 | 5.15 |
| $H_2O$ (8,8) Water ($C_s$) | cc-PVDZ | 1234 | -76.10109262 | 2 | 617 | 0.35 |
| $(CH_3)_2CO$ (20,12) Acetone ($C_{2v}$) | def2-svp | 2186 | -191.89079792 | 2 | 1027 | 0.07 |
| | | | -191.89090301 | 3 | 661 | 0.0082 |
| | | | -191.89089957 | 4 | 455 | 0.01 |
| | | | -191.89089583 | 5 | 340 | 0.01 |
| | | | -191.89079142 | 6 | 391 | 0.08 |
| | | | -191.89079142 | 7 | 384 | 0.08 |
| | | | -191.89089583 | 8 | 343 | 0.01 |
| | | | -191.89079067 | 9 | 209 | 0.08 |
| $(CH_3)_2CO$ (20,12) Acetone ($C_{2v}$) | 6-31G* | 1098 | -192.03451734 | 2 | 513 | 0.08 |
| | | | -192.03456810 | 3 | 304 | 0.04 |
| | | | -192.03451389 | 4 | 208 | 0.08 |
| $(CH_3)_2CO$ (20,12) Acetone ($C_{2v}$) | cc-PVDZ | 1098 | -192.05021581 | 2 | 481 | 0.04 |
| | | | -192.05021210 | 3 | 303 | 0.05 |
| | | | -192.05015569 | 4 | 208 | 0.08 |
| $C_6H_6$ (6,6) Benzene ($D_{6h}$) | cc-PVQZ | 104 | -230.83691596 | 2 | 52 | 0.70 |
| | | | -230.83688457 | 3 | 28 | 0.72 |
| | | | -230.83688457 | 4 | 28 | 0.72 |
| $C_8H_{10}N_4O_2$ (12,9) Caffeine ($C_1$) | sto-3g | 7056 | -667.81482548 | 2 | 3920 | 24.36 |
| | | | -667.81195537 | 3 | 2352 | 26.16 |
| | | | -667.81195537 | 4 | 2352 | 26.16 |
| | | | -667.80878930 | 5 | 784 | 28.15 |
| | | | -667.80878930 | 6 | 784 | 28.15 |
| $Fe(C_5H_5)_2$ (14,10) Ferrocene ($D_{5d}$) | def2-TZVP | 3532 | 1647.01843406 | 2 | 1800 | 0.68 |
| | | | -1647.01800674 | 3 | 1592 | 0.33 |
| | | | -1647.01627361 | 4 | 1052 | 1.42 |

Table S 5: Energy comparison to other methods.

| Method | $N_2$ energy (kcal/mol) | CO energy (kcal/mol) | $H_4O^{2+}$ energy (kcal/mol) | $BH_3$ energy (kcal/mol) | $H_2O$ energy (kcal/mol) |
|---|---|---|---|---|---|
| CISD | -96.35 | -81.18 | -47.68 | -31.08 | -33.64 |
| CISDT | -97.59 | -85.42 | -48.23 | -31.28 | -33.70 |
| CCSD(T) | -103.74 | -88.91 | -49.47 | -31.77 | -34.16 |
| CISDTQ | -105.19 | -88.92 | -49.46 | -31.84 | -34.19 |
| QCD | -105.23 | -89.27 | -49.49 | -31.85 | -34.15 |
| FCI | -105.41 | -89.46 | -49.54 | -31.85 | -34.19 |

Table S 6: Size comparison to other methods.

| Method | $N_2$ size | CO size | $H_4O^{2+}$ size | $BH_3$ size | $H_2O$ size |
|---|---|---|---|---|---|
| CISD | 92 | 162 | 561 | 361 | 49 |
| CISDT | 396 | 790 | 3041 | 1545 | 105 |
| CCSD(T) | 396 | 2173 | 8251 | 3355 | 133 |
| CISDTQ | 1083 | 2173 | 8251 | 3355 | 133 |
| QCD | 396 | 792 | 8820 | 2450 | 65 |
| FCI | 1824 | 3648 | 15876 | 4900 | 133 |

Table S 7: $N_2$ Bond Dissociation.

| Bond Distance | Size | $E_{CL}$ (Hartree) | k | Reduced Size | $\Delta_{FCI-CL}$ (kcal/mol) |
|---|---|---|---|---|---|
| 0.83385 | 1824 | -106.98489234 | 2 | 396 | 0.23 |
| 0.93385 | 1824 | -107.40247938 | 2 | 396 | 0.22 |
| 1.03385 | 1824 | -107.59714601 | 2 | 396 | 0.20 |
| 1.13385 | 1824 | -107.66835022 | 2 | 396 | 0.18 |
| 1.23385 | 1824 | -107.67420956 | 2 | 396 | 0.15 |
| 1.33385 | 1824 | -107.64815357 | 2 | 397 | 0.13 |
| 1.43385 | 1824 | -107.60908805 | 2 | 396 | 0.11 |
| 1.53385 | 1824 | -107.56762396 | 2 | 396 | 0.09 |
| 1.63385 | 1824 | -107.52984467 | 2 | 396 | 0.07 |
| 1.73385 | 1824 | -107.49914796 | 2 | 336 | 0.06 |
| 1.83385 | 1824 | -107.47668893 | 2 | 336 | 0.04 |
| 1.93385 | 1824 | -107.46173680 | 2 | 396 | 0.03 |
| 2.03385 | 1824 | -107.45250906 | 2 | 396 | 0.02 |
| 2.13385 | 1824 | -107.44709122 | 2 | 396 | 0.01 |
| 2.23385 | 1824 | -107.44396273 | 2 | 396 | 0.01 |
| 2.33385 | 1824 | -107.44211517 | 2 | 398 | 0.008 |

Table S 8: $H_3O^+$ Bond Dissociation.

| Bond Distance | Size | $E_{CL}$ (Hartree) | k | Reduced Size | $E_{CL} - E_{FCI}$ (kcal/mol) |
|---|---|---|---|---|---|
| 0.39 | 3136 | -73.21424520 | 2 | 1576 | 9.99e-10 |
| | | -73.21424520 | 3 | 846 | 3.65e-08 |
| | | -73.21424520 | 4 | 846 | 3.65e-08 |
| | | -73.21413644 | 5 | 429 | 0.07 |
| | | -73.21318913 | 6 | 625 | 0.66 |
| 0.59 | 3136 | -74.90123035 | 2 | 1576 | 9.45e-10 |
| | | -74.90123035 | 3 | 846 | 9.70e-07 |
| | | -74.90123035 | 4 | 846 | 9.70e-07 |
| | | -74.90108487 | 5 | 429 | 0.09 |
| | | -74.90108487 | 6 | 429 | 0.09 |
| 0.79 | 3136 | -75.31375455 | 2 | 1576 | 4.99e-10 |
| | | -75.31375455 | 3 | 846 | 2.52e-07 |
| | | -75.31375455 | 4 | 846 | 2.52e-07 |
| | | -75.31361997 | 5 | 429 | 0.08 |
| | | -75.31361997 | 6 | 429 | 0.08 |
| 0.99 | 3136 | -75.39362382 | 2 | 1576 | 1.57e-05 |
| | | -75.39350794 | 3 | 1225 | 0.07 |
| | | -75.39350791 | 4 | 625 | 0.07 |
| | | -75.39350791 | 5 | 625 | 0.07 |
| | | -75.39350791 | 6 | 625 | 0.07 |
| 1.19 | 3136 | -75.37484922 | 2 | 1576 | 1.75e-09 |
| | | -75.37474839 | 3 | 1225 | 0.06 |
| | | -75.37474839 | 4 | 625 | 0.06 |
| | | -75.37477659 | 5 | 925 | 0.04 |
| | | -75.37474839 | 6 | 625 | 0.06 |
| 1.39 | 3136 | -75.32919311 | 2 | 1576 | 5.35e-11 |
| | | -75.32916457 | 3 | 1276 | 0.02 |
| | | -75.32910307 | 4 | 625 | 0.06 |
| | | -75.32910307 | 5 | 625 | 0.06 |
| | | -75.32910307 | 6 | 625 | 0.06 |
| 1.59 | 3136 | -75.28151591 | 2 | 1576 | 9.01e-10 |
| | | -75.28143317 | 3 | 1225 | 0.05 |
| | | -75.28146194 | 4 | 925 | 0.03 |
| | | -75.28146194 | 5 | 925 | 0.03 |
| | | -75.28143317 | 6 | 625 | 0.05 |

Table S 9: $H_3O^+$ Bond Dissociation (cont'd).

| Bond Distance | Size | $E_{CL}$ (Hartree) | k | Reduced Size | $E_{CL} - E_{FCI}$ (kcal/mol) |
|---|---|---|---|---|---|
| 1.79 | 3136 | -75.24069954 | 2 | 1576 | 8.92e-10 |
| | | -75.24062156 | 3 | 1225 | 0.05 |
| | | -75.24065014 | 4 | 925 | 0.03 |
| | | -75.24065014 | 5 | 925 | 0.03 |
| | | -75.24062156 | 6 | 625 | 0.05 |
| 1.99 | 3136 | -75.21042253 | 2 | 1576 | 5.35e-11 |
| | | -75.21034748 | 3 | 1225 | 0.05 |
| | | -75.21037542 | 4 | 925 | 0.03 |
| | | -75.21037542 | 5 | 825 | 0.03 |
| | | -75.21034748 | 6 | 625 | 0.05 |
| 2.19 | 3136 | -75.19122570 | 2 | 1576 | 2.32e-10 |
| | | -75.19119874 | 3 | 1276 | 0.02 |
| | | -75.19117922 | 4 | 925 | 0.03 |
| | | -75.19116894 | 5 | 890 | 0.03 |
| | | -75.19115215 | 6 | 625 | 0.05 |
| 2.39 | 3136 | -75.18083769 | 2 | 1576 | 4.46e-11 |
| | | -75.18081149 | 3 | 1276 | 0.02 |
| | | -75.18079089 | 4 | 925 | 0.03 |
| | | -75.18079089 | 5 | 925 | 0.03 |
| | | -75.18076457 | 6 | 625 | 0.04 |
| 2.59 | 3136 | 75.17583704 | 2 | 1576 | 6.06e-10 |
| | | -75.17576383 | 3 | 1077 | 0.04 |
| | | -75.17576399 | 4 | 781 | 0.04 |
| | | -75.17576383 | 5 | 625 | 0.04 |
| | | -75.17576383 | 6 | 625 | 0.04 |
| 2.79 | 3136 | -75.17359540 | 2 | 1576 | 1.25e-10 |
| | | -75.17359479 | 3 | 1220 | 0.00039 |
| | | -75.17352726 | 4 | 897 | 0.04 |
| | | -75.17352193 | 5 | 625 | 0.05 |
| | | -75.17352193 | 6 | 625 | 0.05 |
| 2.99 | 3136 | -75.17262462 | 2 | 1576 | 3.57e-10 |
| | | -75.17255090 | 3 | 1225 | 0.05 |
| | | -75.17262388 | 4 | 1020 | 0.00046 |
| | | -75.17255090 | 5 | 625 | 0.05 |
| | | -75.17255090 | 6 | 625 | 0.05 |

Table S 10: $H_3O^+$ Bond Dissociation (cont'd).

| Bond Distance | Size | $E_{CL}$ (Hartree) | k | Reduced Size | $E_{CL} - E_{FCI}$ (kcal/mol) |
|---|---|---|---|---|---|
| 3.19 | 3136 | -75.17220417 | 2 | 1576 | 6.06e-10 |
| | | -75.17180634 | 3 | 1184 | 0.25 |
| | | -75.17189117 | 4 | 864 | 0.20 |
| | | -75.16958650 | 5 | 760 | 1.64 |
| | | -75.16947167 | 6 | 584 | 1.71 |
| 3.39 | 3136 | 75.17202395 | 2 | 1576 | 4.19e-10 |
| | | -75.17069160 | 3 | 1176 | 0.84 |
| | | -75.17069162 | 4 | 792 | 0.84 |
| | | -75.17069162 | 5 | 792 | 0.84 |
| | | -75.17069160 | 6 | 584 | 0.84 |

**Derivation for the gauge metric.** In order to derive an expression for a metric function that could be used to gauge the energy difference in the main manuscript, lets consider a Hamiltonian matrix $H$ composed of only two interacting states[1]. That is, we suppose that if state $i$ and $j$ are interacting through $V_{ij}$, the result of their interaction will not be affected by a third state. We have, hence, a collection of so called "two-level systems" represented by the following Hamiltonian matrices:

$$\left\{ \begin{bmatrix} \epsilon_{ii} & V_{ij} \\ V_{ij} & \epsilon_{jj} \end{bmatrix} \right\}_{i \neq j} \tag{S 1}$$

Solving for the eigenvalues we get:

$$\epsilon = \frac{1}{2}(\epsilon_{ii} + \epsilon_{jj}) \pm \frac{1}{2}\sqrt{(\epsilon_{ii} - \epsilon_{jj})^2 + 4V_{ij}^2} \tag{S 2}$$

If we expand this expression up to second order in $V_{ij}$ we get:

$$\epsilon \approx \frac{1}{2}(\epsilon_{ii} + \epsilon_{jj}) \pm \left( \frac{1}{2}|\epsilon_{ii} - \epsilon_{jj}| + \frac{V_{ij}^2}{|\epsilon_{ii} - \epsilon_{jj}|} \right) \tag{S 3}$$

which leads to the following corrections to the states energies:

$$\begin{aligned} \tilde{\epsilon_{ii}} &= \epsilon_{ii} \pm \frac{V_{ij}^2}{|\epsilon_{ii} - \epsilon_{jj}|} \\ \tilde{\epsilon_{jj}} &= \epsilon_{jj} \pm \frac{V_{ij}^2}{|\epsilon_{ii} - \epsilon_{jj}|} \end{aligned} \tag{S 4}$$

and

$$\tilde{\epsilon}_{min} = \min \left( \epsilon_{ii} - \frac{V_{ij}^2}{|\epsilon_{ii} - \epsilon_{jj}|}, \epsilon_{jj} - \frac{V_{ij}^2}{|\epsilon_{ii} - \epsilon_{jj}|} \right) \tag{S 5}$$

This could be extended to all the elements and further corrections by the rest of the stated to obtain the following general expression:

$$\tilde{\epsilon}_{min} = \min_i \left( \epsilon_{ii} - \sum_k \frac{V_{ik}^2}{|\epsilon_{ii} - \epsilon_{kk}|} \right) \tag{S 6}$$

This expression can hence be used to "gauge" for the lowest eigenvalue and by extension, to gauge for the energy difference. Note that this is similar to the Gershgorin method to have a lower/upper

[1]Note that in the graph picture the interacting states will compose the nodes of a graph

bound for the eigenvalues, however, with a different expression for the "discs." To avoid the problem of dividing by 0 when energies are similar, a tunable parameter $\delta > 0$ was added to the denominator leading to the following metric function $f$:

$$f = \min_i \left( \epsilon_{ii} - \sum_k \frac{V_{ik}^2}{|\epsilon_{ii} - \epsilon_{kk}| + \delta} \right) \tag{S 7}$$

If we want an expression without having to introduce a tunable parameter, one can rewrite Eq. S 2 as follows:

$$\epsilon = \epsilon_{ii} + \frac{1}{2}(\epsilon_{jj} - \epsilon_{ii}) \pm \frac{1}{2}\sqrt{(\epsilon_{jj} - \epsilon_{ii})^2 + 4V_{ij}^2} \tag{S 8}$$

which gives us a correction for $\epsilon_{ii}$ provided we pick the right sign on the third term. Let's for now consider the following expression as the "correct" correction of $\epsilon_{ii}$:

$$\tilde{\epsilon}_{ii} = \epsilon_{ii} + \frac{1}{2}(\epsilon_{jj} - \epsilon_{ii}) - \text{sign}(\epsilon_{jj} - \epsilon_{ii})\frac{1}{2}\sqrt{(\epsilon_{jj} - \epsilon_{ii})^2 + 4V_{ij}^2} \tag{S 9}$$

In this case, if $\epsilon_{jj} - \epsilon_{ii} > 0$, the expression becomes:

$$\tilde{\epsilon}_{ii} = \epsilon_{ii} + \frac{1}{2}(\epsilon_{jj} - \epsilon_{ii}) - \frac{1}{2}\sqrt{(\epsilon_{jj} - \epsilon_{ii})^2 + 4V_{ij}^2} \tag{S 10}$$

which tends to $\epsilon_{ii}$ when $V_{ij}$ tends to 0 since the second term is positive. On the other hand, when $\epsilon_{jj} - \epsilon_{ii} < 0$, the expression becomes:

$$\tilde{\epsilon}_{ii} = \epsilon_{ii} + \frac{1}{2}(\epsilon_{jj} - \epsilon_{ii}) + \frac{1}{2}\sqrt{(\epsilon_{jj} - \epsilon_{ii})^2 + 4V_{ij}^2} \tag{S 11}$$

which also tends to $\epsilon_{ii}$ when $V_{ij}$ tends to 0 since the second term is negative.

We can now think about another expression for the metric gauging the energy difference which will read as follows:

$$g = \min_i \left( \epsilon_{ii} + \frac{1}{2} \sum_k \left( (\epsilon_{ii} - \epsilon_{jj}) - \text{sign}\sqrt{(\epsilon_{ii} - \epsilon_{jj})^2 + 4V_{ij}^2} \right) \right) \tag{S 12}$$

which has the following equivalent expression[2]:

$$g = \min_i \left( \epsilon_{ii} + \frac{1}{2} \sum_k \left( |\epsilon_{ii} - \epsilon_{jj}| - \sqrt{(\epsilon_{ii} - \epsilon_{jj})^2 + 4V_{ij}^2} \right) \right) \tag{S 13}$$

Although Eq. S 13 is more rigorous since it does not possess any tunable parameter, it is also computationally more demanding since more operations need to be performed. This is an

[2]This can be easily proven considering the cases when $\epsilon_{jj} > \epsilon_{ii}$ and vice-versa

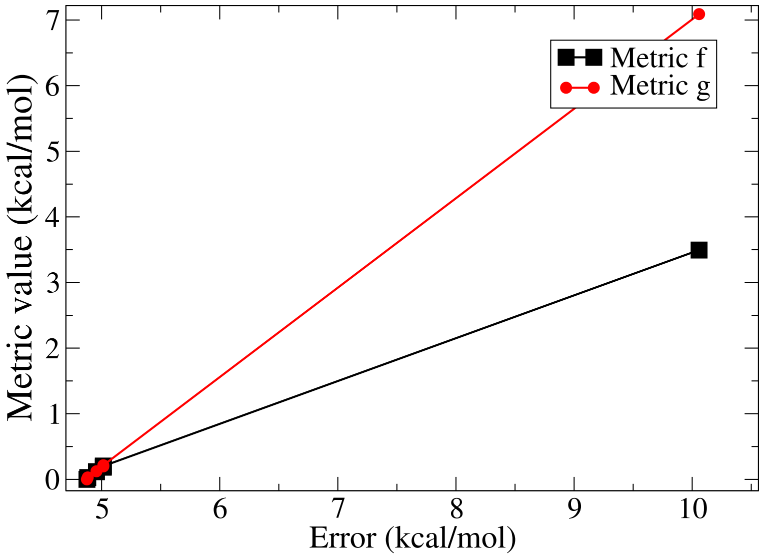


Figure S 2: Comparison between metric $f$ and $g$ in their ability to gauge the energy difference. The metric values were referred to the *2-clustering* value to ease comparison. The system used here is a CO molecule FCI Hamiltonian matrix in the sto-3g basis set.

important point to consider since metric $f$ is used as a gauge for the energy difference and it will need to be computed for every cluster at every *k-clustering* on a classical computer. This is hence one of the reasons for using metric $f$ instead of $g$ to gauge for the energy difference in this work.

Fig. S 2 shows a comparison of both metrics on the k-clustering of molecule CO using the FCI Hamiltonian matrix with sto-3g basis set. A parameter of $\delta = 1$ was used for computing metric $f$. We can clearly see that both metrics agree when the energy differences are low whereas metric $f$ deviates from the linear trend as compared to metric $g$ when the energy differences are higher. For the purpose of gauging the energy difference they are both equally suitable with the caveat that $f$ is simpler to compute.

As was shown in the main manuscript, the metric shows both a good monotonicity and a good linearity for all the cases analyzed. It can determine the relevant cluster (without having to compute the energies for all the sub-graphs from the k-clustering). The sub-graph with the lowest gauge metric value will lead to the lowest energy (lower energy difference). Moreover, one of the advantages of this metric is that the formula is simple enough to understand what conditions for the cluster lead to the lowest energy. Here we have identified three main conditions:

- The lower the diagonal elements that are picked in the partition (relevant cluster), the lower will be the energy.
- The closer the energies of the nodes that belong to the partition that is picked, the lower will

Table S 11: Chemical accuracy from gauge space.

| Molecule | Size | $\text{Gauge}_{FCI}$ (Hartrees) | $\text{Gauge}_{CL}$ (Hartrees) | Reduced Size | $E_{CL} - E_{FCI}$ (kcal/mol) |
|---|---|---|---|---|---|
| $H_2$ 631G* | 8 | -1.86775811 | 0.00870209 | 4 | 5.56 |
| $H_2$ cc-PVDZ | 22 | -1.86093991 | -1.85059347 | 12 | 6.49 |
| $H_2$ 6-311++G** | 54 | -1.87796529 | -1.87382138 | 29 | 2.60 |
| $H_2$ aug-cc-PVQZ | 1256 | -1.88353813 | -1.87335889 | 492 | 6.39 |
| $H_2O$ sto-3g | 133 | -83.91116169 | -83.91106565 | 65 | 0.06 |
| $BH_3$ sto-3g | 1250 | -33.73576371 | -33.73297808 | 625 | 1.75 |
| | | | -33.73526023 | 321 | 0.31 |
| $N_2$ sto-3g | 1824 | -130.5234671 | -130.5181132 | 1036 | 3.36 |
| | | | -130.5127594 | 648 | 6.72 |
| | | | -130.5180307 | 544 | 3.41 |
| | | | -130.5230616 | 396 | 0.25 |
| HCN (8,10) sto-3g | 1576 | -115.369203 | -115.369203 | 792 | 1.25e-05 |
| HCN (9,10) 6-31G* | 4076 | -117.3139781 | -117.3063941 | 2110 | 4.76 |
| $C_6H_6$ (6,6) cc-PVQZ | 104 | -436.2190515 | -436.2182797 | 52 | 0.48 |
| $C_8H_{10}N_4O_2$ (12,9) sto-3g | 7056 | -1584.331104 | -1584.316145 | 3920 | 9.38 |

be the energy difference.

- The higher the coupling between nodes, the lower will be the energy difference.

This metric, together with the results presented, can tell us when the clustering method will work better. From the expression of $f$ we can conclude that the clustering method will work better when the couplings between nodes are high as compared to the other conditions (proximity of the state energies in the relevant cluster, and the degree of how low are the individual energies of the relevant cluster).

The gauge can also be used to indicate whether a low energy cluster will be within chemical accuracy before diagonalization. The difference between the gauge of the original matrix and the gauge of the cluster will be within chemical accuracy ($\leq$ 1.0 kcal/mol or $\leq$ 1.6e-03 Hartrees). This is shown for a number of molecules in Table S 11. $H_2$ molecule results were not shown previously. The others were previously seen in Table S 1 and Table S 4. All molecule clusters that resulted in chemical accuracy were predicted correctly by using the gauge. Additionally, the gauge indicates when a cluster will not result in chemical accuracy. This could indicate that the original matrix could not be reduced using *Quantum Community Detection* or was already optimal. Further analysis is required to understand this fully.

## References

1. Parrish, R. M. *et al.* Psi4 1.1: An open-source electronic structure program emphasizing automation, advanced libraries, and interoperability. *J. Chem. Theory Comput.* **13**, 3185–3197 (2017).
